# Influence of Hybridization on Tensile Behaviors of Non-Absorbable Braided Polymeric Sutures

**DOI:** 10.3390/polym12030682

**Published:** 2020-03-19

**Authors:** Moqaddaseh Afzali Naniz, Mahdi Bodaghi, Majid Safar Johari, Ali Zolfagharian

**Affiliations:** 1 Department of Engineering, School of Science and Technology, Nottingham Trent University, Nottingham NG11 8NS, UK; afzali@aut.ac.ir; 2 Department of Textile Engineering, School of Material Engineering & Advanced Processes, Amirkabir University of Technology, Tehran 158754413, Iran; mjohari@aut.ac.ir; 3 School of Engineering, Deakin University, Geelong, Victoria 3216, Australia; a.zolfagharian@deakin.edu.au

**Keywords:** braided composite sutures, hybridization, non-absorbable, mechanical performance, synthetic polymers, experiment

## Abstract

This paper aims to investigate the effects of fiber hybridization technique on the mechanical behaviors of non-absorbable braided composite sutures. Fifteen types of hybrid braided sutures (HBSs) made of polyester (PET), polypropylene (PP), and polyamide 6 (PA6) are produced and tested to measure ultimate tensile strength (UTS), maximum strain, elastic modulus, and breaking toughness. Based on the results, it is observed that the suture material plays a significant role in the tensile and mechanical performance of HBSs, and they can be tailored through the different combinations of yarns according to the required mechanical properties. Experiments exhibit occurrence positive hybrid effect in both maximum strain and elastic modulus, and negative hybrid effect in UTS. The optimal tensile performance is associated with the hybrid structure comprising 75% PA6-12.5% PET-12.5% PP. This means the ternary structure with higher PA6 content along with PP and PET, demonstrates a synergistic effect. Thus, such a ternary composite structure is very promising for the design of novel non-absorbable sutures. Due to the absence of similar results in the specialized literature, this paper is likely to advance the state-of-the-art composite non-absorbable sutures and contribute to a better understanding of the hybridization concept for optimizing composite material systems.

## 1. Introduction

Polymeric sutures have a prominent role among all other medical implants and are described as strands of materials used for closure of wounds caused by surgery or trauma and consequently improve wound healing [1]. They are, in general, made up of fibers from natural or synthetic polymers, in which polymeric fibers could be absorbable or non-absorbable [2,3]. Accordingly, sutures are commonly categorized as absorbable and non-absorbable materials since the former loses its tensile strength within a defined time scale, while the latter remains almost unchanged by biological activities of body tissues [4,5]. This classification indicates that the compatibility of a suture is extensively associated with its mechanical performance characteristics, e.g., the tensile strength [6,7]. Non-absorbable sutures are monofilaments or multifilament structures generally composed of polyamide (PA), polyester (PET), or polypropylene (PP), which have played an essential role in the advancement of surgical procedures due to their excellent mechanical properties, such as tensile strength, fatigue resistance, and high flexibility [8,9]. 

Multifilament sutures regularly comprise several filaments or strands twisted or braided together, offering greater tensile strength, pliability, and flexibility in comparison to monofilament sutures [10,11]. In other words, although braided sutures may not have particular advantages over monofilament sutures in terms of wound healing properties, they have outstanding handling properties and flexibility that outweigh the beneficial healing properties of monofilament sutures. Braided multifilament sutures are known for their exceptional mechanical properties in their longitudinal directions, which can be manufactured on a circular braiding machine [12,13]. Circular braiding is an ancient technique for manufacturing tubular structures by interlacing three or more strands of yarns in such a way that they cross one other, and each set of strand forms a well-defined braid pattern following the helical path. In a braided structure, the mechanical properties can be variously designed according to the requirements, as various braiding patterns (e.g., diamond, regular, and Hercules), axial configurations (biaxial and triaxial), braid diameters, and braiding angles are attainable [14,15,16,17,18].

Considering there is no single suture material which can fulfill all the essential requirements of sutures (i.e., not breaking unexpectedly during the service, elongating with wound edema, being biocompatible, handling efficiently, and forming a secure knot), it is most important to select the optimal suture material to ensure the best possible outcome for surgery [19,20,21]. Hybridization is among the most effective techniques by which the structure characteristics can be modified. On that account, the fiber hybridization is considered a potential solution for optimizing structures concerning the requirements [22,23]. Hybrid braids are usually considered as structures that combine two or more types of fibers offering a broad range of properties that cannot be obtained by a single kind of fiber [24]. They are of interest when the desired mechanical response of the structure requires qualities available only from different types of yarn; for example, stiffness combined with energy absorption, or a nonlinear elastic response [25,26].

The prediction of the tensile property is a prerequisite for the successful deployment of the braided structures in suturing [9,15,27]. Although several attempts have been made to investigate tensile properties and mechanical performance of braided sutures [28,29,30,31,32,33,34,35,36], as far as the authors are aware, no scientific work that encompasses the scope of fiber hybridization on the mechanical behavior of braided sutures can be found. 

In this study, the possibility of using fiber hybridization technique for the development of new types of non-absorbable composite sutures with the highest tensile performance was explored. Thus, the present study aimed to determine an optimal combination of three most common non-absorbable suture materials toward manufacturing the structures permitting to attain this purpose. Accordingly, fifteen sets of hybrid braided sutures (HBSs) consisting of the most common fibers in non-absorbable sutures, e.g., PET, PP, and PA6, were designed and manufactured on a circular braiding machine. To demonstrate the HBSs’ mechanical properties, tensile test was carried out. A statistical analysis of variance (with the help of the SPSS Statistics) was applied to examine the experimental results and their meaningful differences. Additionally, the corresponding mechanical parameters were scored in order to specify the hybrid sutures featuring desirable tensile performance. The results provided in this paper are expected to be instrumental in designing efficient and reliable non-absorbable sutures.

## 2. Materials and Methods

### 2.1. Materials

The non-texturized PET, PP (with high extension at failure), and PA6 (which enhances tensile strength and modulus of elasticity) with an identical yarn fineness of 200 den supplied respectively by (IRAN TAK NAKH CO., Nowshahr, Iran), (Behkoosh Industrial Group, Isfahan, Iran), and (ALIAF P.J.S CO., Tehran, Iran) were used for manufacturing various HBSs.

### 2.2. Tensile Properties of Yarns

The yarns’ tensile properties were characterized by conducting force-displacement tests on an Instron TM/SM (Instron, High Wycombe, UK) universal testing machine as per the ASTM D2256/D2256M_2010 [37]. The mean and standard deviation of the experimental results from ten trials per sample are listed in Table 1.

### 2.3. Suture Fabrication

**Figure 1:**
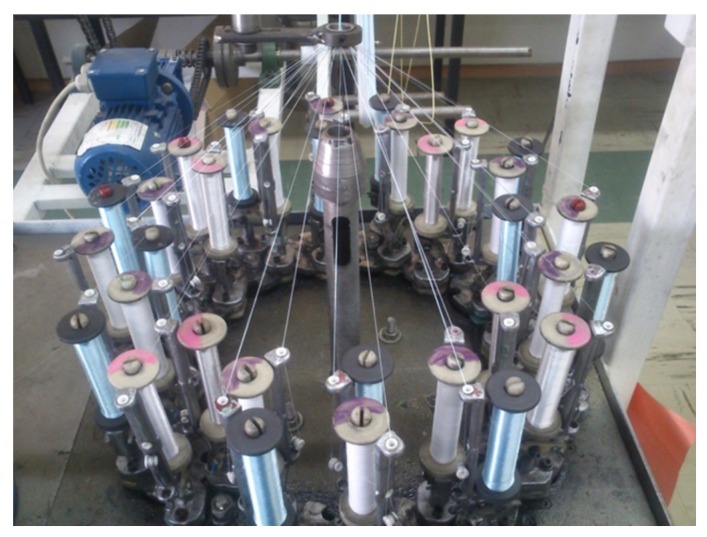
32-carrier vertical braiding machine used for manufacturing of sutures.

**Figure 2:**
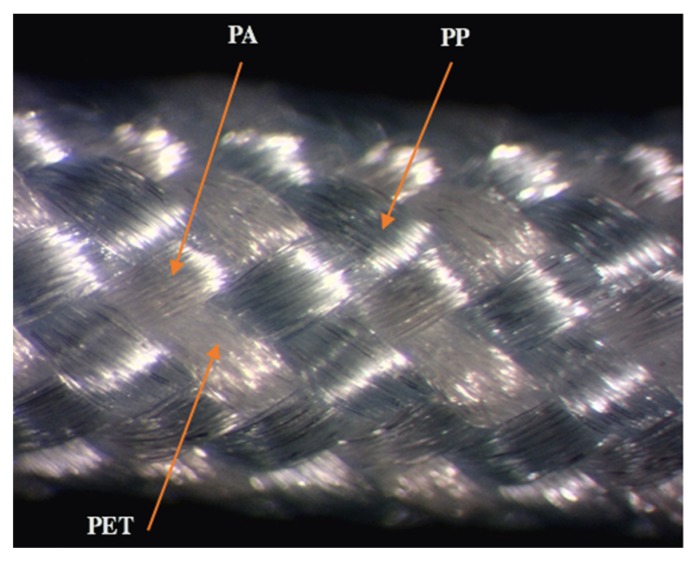
Microscope images of sutures’ surface texture (magnification, 25×).

The HBSs with an unvarying diameter of 2 ± 0.1 mm made up of 32 multifilament yarns were fabricated on a thirty-two-carrier vertical braiding machine (Gole-Narges Co., Yazd, Iran) as shown Figure 1. Sutures were braided in a regular braid pattern to achieve small diameters and smooth surfaces [31]. The surface texture of HBSs is depicted in Figure 2. A fixed ratio (2:20) of the take-up speed to the carrier resulted in a braiding angle of 40 ± 1°. Braid diameters and braid angles were determined through taking images of braids and then analyzing them by a low magnification microscope. Then, photos were investigated by ImageJ (1.50e, National Institutes of Health, Bethesda, MD, USA). Five meters length of braided sutures were observed under the microscope, and an average of fifty readings was recorded to measure the braid diameter and braid angle [38].

The same scheme was used to fabricate other braids, featuring different fiber compositions as listed in Table 2. The number of each thread divided by the total 32 yarns gave the percentage of fiber content in each structure. It should be noted that, in a hybrid structure, each yarn has a different mechanical tensile characteristic, in which higher tension of some of the yarns could result in a spiral effect. Therefore, the symmetrical distribution of yarns was considered while designing the structures to avoid this problem.

Ideally, suture’s diameter should follow the United States Pharmacopeia (USP) classification of suture size [39]; though, many commercial sutures are clearly labeled that they do not correspond to the USP diameter standards [40]. In the current study, the suture diameter is about 2 ± 0.1 mm, which is higher than the standard and affects the calculated material properties such as failure stresses of the sutures due to the inverse relationship between stress and diameter. Hence, providing a comparison in the obtained results of the current study with those in the literature is not practical. However, it should be considered that the goal of this research is to investigate the hybridization effect on the mechanical properties of braided sutures and to determine an optimal combination of PP, PET, and PA6 toward manufacturing the structures with the highest tensile performance.

### 2.4. Tensile Characteristics of Sutures

**Figure 3:**
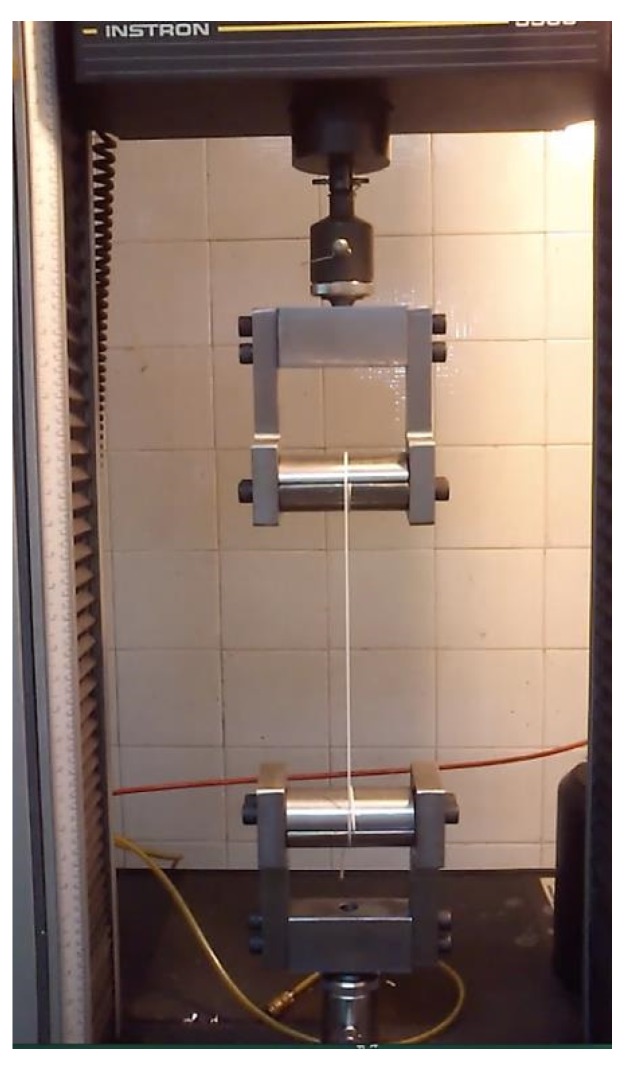
Tensile strength test using Instron tensile testing machine.

The tensile characteristics of the braids were obtained by force-displacement tests carried out by means of an Instron TM/SM (Instron, High Wycombe, UK) universal testing machine following the ASTM D6775-13 [41], as shown in Figure 3. It includes determination of the tensile strength and tensile strain of braided structures with the help of a split-drum type specimen clamp. Thus, a specific jaw was designed and manufactured as per the standard approach [42]. The experiments were conducted under standard conditions for relaxed specimens, i.e., 21 ± 1 °C temperature, 65 ± 2% relative humidity, a crosshead speed of 75 mm/min, and a gauge length of 250 mm. All the braid types underwent these tests for ten times. The means and standard deviations of the ten-trial test results were also calculated. It should be stated that all the sutures were conditioned at least 24 h before tests to bring them to the moisture equilibrium in the specified atmosphere in which the testing was to be performed. Moreover, regarding that many of the physical properties of textile products are influenced by relative humidity and temperature in a manner that affects the results [43], all the tensile tests were done on the same day to make reliable comparisons among different structures.

The mode and the location of failure of the specimens were recorded as the tensile tests were conducted. All the specimens showed brittle failure, failed at the gage area, and a large number of them failed near the center. For that reason, all of the observed failure modes were deemed valid, based on which the calculation of mechanical properties of the specimens was performed. 

### 2.5. Statistical Analysis 

A descriptive statistical analysis was carried out in the present study. Significance was assessed at a 5% level. A one-way analysis of variance (ANOVA) was used to assess the tensile strength of braided sutures at different combinations. Accordingly, all the mechanical properties data were assessed one by one in order to evaluate the significance of the experimental results. Furthermore, Duncan’s multiple range test (MRT) identified the contrasting HBS’s tensile properties. Finally, applying the Post-Hoc test, ten out of fifteen structures were chosen to analyze the hybridization effect on tensile behaviors. Statistical software, namely SPSS ver. 15.0 (SPSS Inc., Chicago, IL, USA) were used for the analysis of the data.

## 3. Results and Discussion

The primary purpose of this study was to develop a special class of braided sutures-hybrid for enhanced mechanical and tensile performance. This novel method was inspired by the hybridization of the reinforcement fabrics in composites, which compared to non-hybrid systems, allows for integrating beneficial features of different fiber systems and well-balanced mechanical properties.

**Figure 4:**
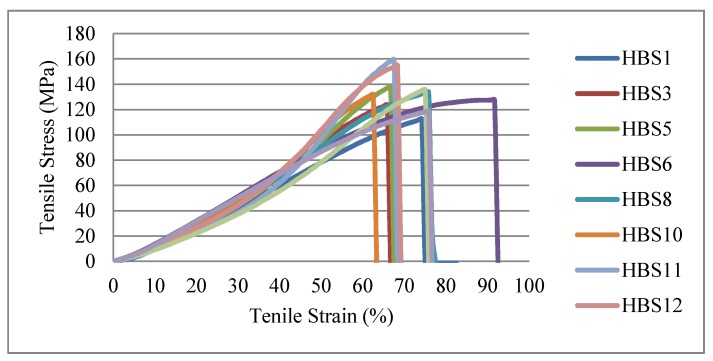
Stress–strain curves of braided sutures.

Most published articles on the tensile behavior of various sutures focus solely on the breaking force. Comprehensive reports and analyzing other important tensile properties such as failure strain and stress, elastic modulus, and full stress–strain curves across suture materials are quite limited [40]. The current study presents stress–strain data to illustrate differences in the sutures that can be attributed to the material type and their combinations. Figure 4 shows the stress–strain curves for non-absorbable suture materials. The composition of fibers plays a key role in the behavior of the developed HBSs under a certain tensile loading. Besides, it can be seen the initial region of diagrams has a low slope due to the change of yarn path in the braid structure. In other words, at low load levels, braids go through a geometric transition, and there is practically no elastic deformation in the yarn. At higher tensile loads, the rearranged yarns reduce the braid diameter. When the load increases more, the braid begins to reach a jammed state. In the jamming condition, the decrease in the diameter of the braid is almost negligible, and the yarn properties direct the mechanical response. Hence, the braid response under tensile load can be analyzed into two steps. First, the geometry of the braid is changed, and the yarns are aligned with the applied force (jamming condition). Second, the yarns are extended, and their mechanical property presents the main role. This observation is in good accordance with findings by previous studies that investigated the tensile properties of tubular braids [38,42]. 

### 3.1. Maximum Strain

**Figure 5:**
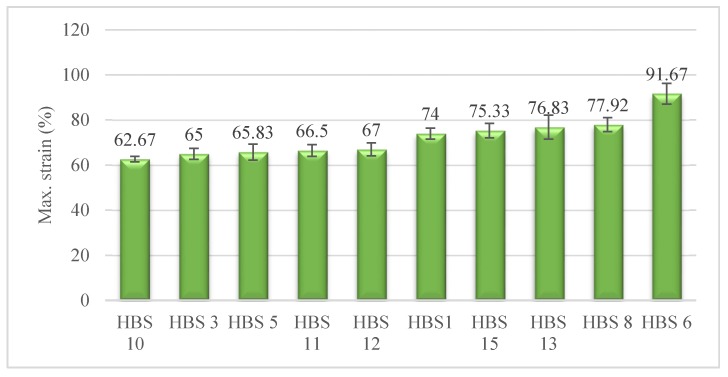
Average maximum tensile strain of HBSs.

Figure 5 illustrates the maximum strain of HBSs. From the results presented in this figure, it can be seen that HBS6 has superior tensile strain. This observation was anticipated since the PP has the highest tensile strain among the other two fibers, see Table 1. Increased elongation would be advantageous in situations where a great deal of edema is expected postoperatively [44]. 

The following three groups are suggested for the hierarchy of maximum tensile strain with reference to the maximum strain of HBSs as well as Duncan’s test results:

(HBS10, HBS3, HBS5, HBS11, HBS12) < (HBS1, HBS15, HBS13, HBS8) < HBS6

**Figure 6:**
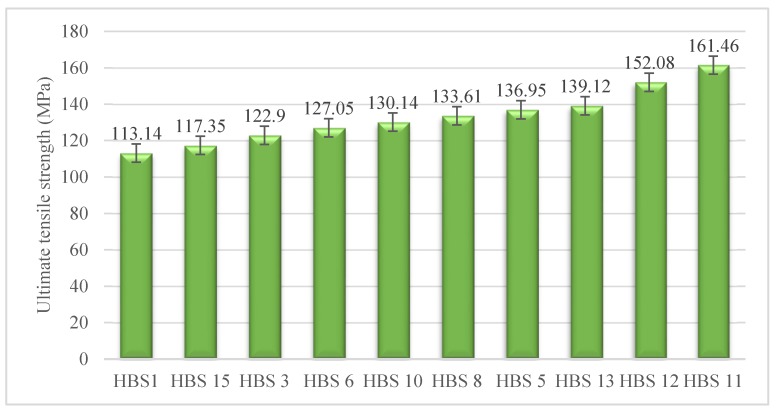
Average ultimate tensile strength of HBSs.

The closeness of the HBSs’ tensile strain values in a group helps to choose the right structure featuring the specific properties. As a case in point, HBS12 (75% PA6-12.5% PP-12.5%PET) and HBS3 (50% PET-25% PP-25% PA6) with the same maximum strain, yield different tensile strengths as can be seen in Figure 6. Thus, it is possible to have a higher tensile strength at the same value of tensile strain by replacing HBS3 with HBS12.

By focusing on the results in Figure 5, an unanticipated finding can be concluded that the maximum strain of HBS13 (50% PA6-25% PP-25% PET) is superior to that of HBS5 (50% PA6-50% PP). More precisely speaking, the hybrid structure consisting of a 25% lower amount of PP gives about 16.7% higher maximum strain. However, it should be noted that PP has about 5.4% higher maximum strain than PET. The cause for this is not entirely obvious; however, it may have something to do with the ‘hybrid effect’ that is associated with the interaction in a hybrid structure. This phenomenon is explained as the deviation of a hybrid structure from the rule of mixture [45], or the difference between the performance of fiber in a hybrid structure and non-hybrid [46].

Additionally, by comparing HBS8 (50% PP-25% PET-25% PA6) with HBS3 (50% PET-25% PA6-25% PP-), it can be revealed that the replacement of 25% of PET fibers with PP resulted in 19.9 % improvement in the value of tensile strain. This finding is in agreement with previously reported results and could be justified as follows. When it comes to a hybrid structure, the more the strain at failure, the greater the ratio of less-stiff higher-elongation fiber (LSHEF) to more-stiff lower-elongation fiber (MSLEF). The higher amounts of LSHEFs in the structure at higher quantities make it possible for the structure to bear the redistributed loads even beyond the breakage of MSLEFs. Furthermore, it is likely for the loads to be transferred back to the broken MSLEFs and partially sustained through a positive hybrid effect. This results in greater breaking strength and strain [47,48,49]. Considering the value of maximum strain for HBS10 (50% PA6-50% PET) and HBS5 (50% PA6-50% PP), it can be concluded that when the LSLEFs presence in these hybrid structures had almost equal quantity to those of the MSLEFs, they could not bear the load sustained by the structure after the breakage of MSLEFs. This resulted in catastrophic failure and poor tensile properties [49,50].

### 3.2. Ultimate Tensile Strength

The tensile strength of suture materials has been identified as critical to secure suturing [40]. Figure 6 shows the average ultimate tensile strength (UTS) of HBSs. The highest UTS is observed in the cases of HBS11 and HBS 12. It is apparent that the number of PA6 fibers in the combination has a major impact on tensile strength, which is due to the more tensile strength of PA6 than those of two other threads, see Table 1. In other words, since these structures are the richest in PA6 fibers with a superior tensile strength, they are able to tolerate heavier loads compared to the other structures.

Statistically significant differences in the UTS of HBSs are suggested by the analysis of variance. Below, the hierarchy of HBSs are divided into eight groups based on the Duncan’s test results:

HBS1 < HBS15 < HBS3 < (HBS6, HBS10) < HBS8 < (HBS5, HBS13) < HBS12 < HBS11

It can be inferred from Figure 6, that HBS8 (50% PP-25% PA6-25% PET) yielded greater tensile strength than HBS3 (50% PET25% PA6-25% PP), and the P-values indicate a statically significant difference; however, an opposite result was anticipated with regard to the tensile characteristics of the threads in their structures (Table 1). The reason for this is not clear; however, it may be associated with the hybrid effect that is connected with the interaction in a hybrid structure. This phenomenon can be considered as the difference in the performance of the fiber in hybrid and non-hybrid structures, which influenced by the relative volume fraction of components, and can have positive or negative values [51,52].

### 3.3. Elastic Modulus

**Figure 7:**
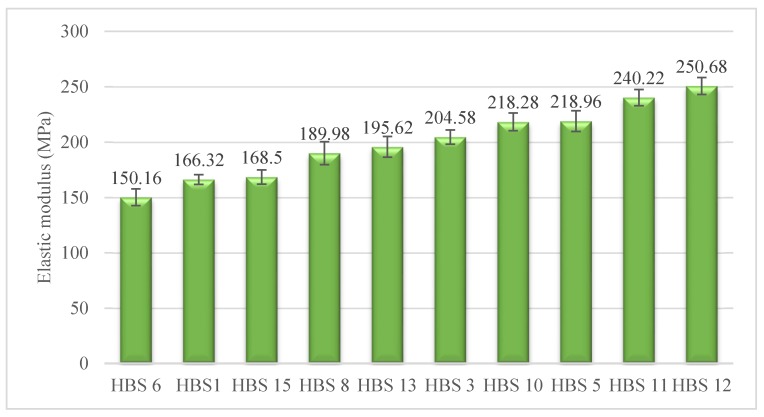
Average elastic modulus of hybrid braided sutures (HBSs).

Figure 7, namely the elastic modulus of HBSs, implies that an increase in HBSs’ modulus is proportional to the number of fibers with a higher modulus. One of the interesting results from our studies on tensile behavior is that HBS12 exhibited the highest elastic modulus. This means that the ternary combination of 75% PA6-12.5% PET-12.5% PP led to a positive hybrid effect in elastic modulus [46].

Analysis of variance shows that there are statistically meaningful differences among the elastic modulus of HBSs. The Duncan’s test results help to categorize the hierarchy of HBSs into seven groups:

HBS6 < (HBS1, HBS15) < (HBS8, HBS13) < HBS3 < (HBS10, HBS5) < HBS11 < HBS12

Another important finding was that the elastic modulus of HBS11 is about 61% higher than that of HBS6, while the elastic modulus of PA6 fibers is about 39% higher than that of PET fibers, and it is contradictory to what is expected from the data in Table 1. Similarly, the P values show that there are statistically significant differences between the modulus values of these structures. A possible explanation for this inconsistency might be that HBS6 with a more extensible structure (about 38%), has lower resistance to an extension for small extensions [53], which, consequently, results in a lower elastic modulus. A similar phenomenon is observed for HBS11 and HBS1, which a possible explanation for that could be the difference in the extensibility of their structures.

### 3.4. Breaking Toughness

**Figure 8:**
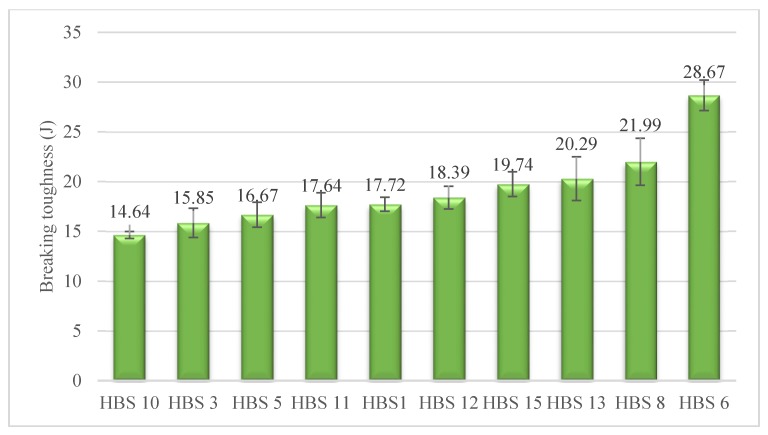
Average breaking toughness of HBSs.

The area below the stress–strain diagram represents the work done the specimen is extended to the breaking point. In other words, it can be considered as the energy absorbed by the structure per unit volume up to rupture [54]. On that account, the stress–strain curves of HBSs were examined employing the OriginPro 8.6, to determine the breaking toughness. Average breaking toughness of HBSs is shown in Figure 8, in which HBS6 delivers the greatest failure energy on account of its high tensile strain. Comparing Figure 8 to Figure 5 reveals that both of them present a similar pattern and absorbed energy increased by an increase in the tensile strain of the constituent fibers. Thus, to sum up, there is a direct relationship between the tensile strain and the total absorbed energy before the rupture takes place. 

Analysis of variance showed that there were statistically significant differences in the results. Given the tensile strain of HBSs, and based on the Duncan’s test results, the hierarchy of the tensile strain can be classified into six groups as follows:

HBS10 < (HBS3, HBS5) < (HBS11, HBS1) < HBS12 < (HBS15, HBS13, HBS8) < HBS6

### 3.5. Ranking HBSs as Regards Their Tensile Performance

The Duncan’s test results made it possible to categorize the hierarchy of HBSs for each evaluated parameter, see Table 3. Suture materials are ordered by increasing the value of each property from left to right, and the ones in each parenthesis have insignificant differences.

A ranking criterion is defined to identify the hybrid suture with the most satisfactory tensile performance. For that purpose, a score in each of the evaluated parameters—maximum strain (%), ultimate tensile strength (MPa), and modulus of elasticity (MPa) is established in Table 4. It should be recognized that HBSs that are in a category (i.e., with insignificant differences) have the same score for each parameter. Accordingly, the one with the highest total score delivers the peak tensile performance. 

According to Table 4, HBS12 and HBS11 present the best tensile performance, whereas HBS1 brings the lowest one. Although HBS11 and HBS12 reveal the same total scores, they have remarked differences in the physical properties and fiber combination which influence their mechanical behavior. It has been found that PA sutures have poor handling and knot security while PET sutures reveal good handling, and PP sutures easily pass through tissues [9]. For instance, it can be concluded that in HBS12, the PA6 provides mechanical strength, while the PET improves handling, and PP helps the structure easily pass through tissues. Moreover, because of the inherent susceptibility of the amide linkage to hydrolytic degradation, PA sutures have been reported to lose strength after implantation. Therefore, in terms of knot strength performance (i.e., minimum change in strength over the implantation time), HBS12 is expected to have better performance since PET and PP are not affected by water [55,56]. To be more specific, PET has hydrocarbon backbones, which contain ester linkages and are hydrophobic, while PA6 is affected by water because of the amide groups in its backbone chain, which are highly polar and can form a hydrogen bond with water easily [57].

It can be concluded that in order to exploit fibers in the suture structure, the design of materials and structures is a major issue that needs to be addressed. Moreover, the dramatic difference in the mechanical properties of fibers cause the variation of the hybridization effects on hybrid structures. Thus, appropriately using a hybrid design would help to overcome hybridization challenges in fabricating hybrid braided composite sutures.

## 4. Conclusions

In the present work, the hybridization technique was introduced to the non-absorbable suture manufacturing process based on variation in fiber type and combination. This method, inspired by the hybridization of the reinforcement fabrics in composites, aimed to improve the tensile strength of the braided sutures and secure it after suturing. Accordingly, a special class of braided sutures (i.e., hybrid sutures) formed from three distinct polymeric yarn types in a single structure. Therefore, a practical experimental study for investigating the hybridization effect has been done based on the determination of load-extension curves. All the mechanical properties data were evaluated individually to find out whether the contribution of fiber hybridization to tensile behavior was significant. It was discovered that the greatest the amount of PA6 fibers in the HBSs, the better tensile performance and mechanical properties.

Moreover, with the defined ranking criterion taken into account, a ternary hybrid suture with a combination of 75% PA6-12.5% PET-12.5% PP presented the greatest values of tensile performance among hybrid structures. Results of the tensile test implied that there was a positive hybrid effect in maximum strain and elastic modulus, and a negative hybrid effect in UTS for some of the studied HBSs. However, no hybrid effect for breaking toughness was found. Additionally, results revealed that the suture material (PP, PET, and PA6), and their combination has a direct bearing on tensile performance, and also the nature of the ultimate behavior could be influenced by the interaction within each pair of constituent fibers. It was concluded that the hybridization of PA6 fiber with PET and PP fibers at a proper combination would offer a structure with higher tensile performance. Lack of similar findings in other pieces of research makes this paper a practical work when it comes to the fabrication of non-absorbable braided composite sutures. It also provides a baseline for the researchers who will deal with fabrication and composite material systems designed specifically for use in optimized structural concepts.

## Figures and Tables

**Table 1:** Characteristic of yarns used for experiments.

Fiber Type	Number of Filament Per Yarn	Tensile Strength (MPa)	Strain at Break (%)	Elastic Modulus (MPa)	Breaking Toughness (MPa)
Polypropylene	72	420.75 ± 8.45	21.36 ± 0.86	4145.34 ± 121.97	53.65 ± 3.81
Polyamide 6	34	692.68 ± 21.51	18.82 ± 0.99	5740.74 ± 143.37	55.12 ± 9.53
Polyester	72	483.51 ± 24.63	20.25 ± 0.53	5890.81 ± 229.86	59.54 ± 8.45

**Table 2:** Specifications of manufactured sutures.

Braid ID	Fiber (%)		
	Polyamide 6	Polypropylene	Polyester
HBS.1	0	0	100
HBS.2	12.5	12.5	75
HBS.3	25	25	50
HBS.4	37.5	37.5	25
HBS.5	50	50	0
HBS.6	0	100	0
HBS.7	12.5	75	12.5
HBS.8	25	50	25
HBS.9	37.5	25	37.5
HBS.10	50	0	50
HBS.11	100	0	0
HBS.12	75	12.5	12.5
HBS.13	50	25	25
HBS.14	25	37.5	37.5
HBS.15	0	50	50

**Table 3:** HBSs order based on Duncan’s test results in each evaluated tensile parameter.

Tensile Parameter	Hierarchy of HBSs
Maximum strain	(HBS10, HBS3, HBS5, HBS11, HBS12) < (HBS1, HBS15, HBS8, HBS13) < HBS6
Ultimate tensile strength	HBS1 < HBS15 < HBS3 < (HBS6, HBS10) < HBS8 < (HBS5, HBS13) < HBS12 < HBS11
Elastic modulus	HBS6 < (HBS1, HBS15) < (HBS8, HBS13) < HBS3 < (HBS10, HBS5) < HBS11 < HBS12
Breaking Toughness	HBS10 < (HBS3, HBS5) < (HBS11, HBS1) < HBS12 < (HBS15, HBS13, HBS8) < HBS6

**Table 4:** Score board of HBSs.

Braid ID	Maximum Strain (%)	Ultimate Tensile Strength (MPa)	Elastic Modulus (MPa)	Total Score
HBS.1	2	1	2	5
HBS.3	1	3	4	8
HBS.5	1	6	5	12
HBS.6	3	4	1	8
HBS.8	2	5	3	10
HBS.10	1	4	5	10
HBS.11	1	8	6	15
HBS.12	1	7	7	15
HBS.13	2	6	3	11
HBS.15	2	2	2	6

## References

[1] Joseph B., George A., Gopi S., Kalarikkal N., Thomas S. (2017). Polymer Sutures for Simultaneous Wound Healing and Drug Delivery—A Review. Int. J. Pharm..

[2] Pillai C.K.S., Sharma C.P. (2010). Review paper: Absorbable polymeric surgical sutures: Chemistry, production, properties, biodegradability, and performance. J. Biomater. Appl..

[3] Visco A., Scolaro C., Giamporcaro A., De Caro S., Tranquillo E., Catauro M. (2019). Threads made with blended biopolymers: Mechanical, physical and biological features. Polymers.

[4] Rajendran S., Anand S.C. (2002). Developments in medical textiles. Text. Prog..

[5] Gokarneshan N., Velumani K. (2018). Recent Innovations in Textile Sutures—An Approach towards Improved Surgical Procedures. Sci. J. Biomed. Eng. Biomed. Sci..

[6] Chellamani K.P., Veerasubramanian D., Balaji R.S.V. (2013). Surgical Sutures: An overview. J. Acad. Indus. Res..

[7] Greenberg J.A., Clark R.M. (2009). Advances in Suture Material for Obstetric and Gynecologic Surgery. Rev. Obstet. Gynecol..

[8] Traoré A.S., Guidoin M.F., Marois Y., Zhang Z., Douville Y., Guidoin R., King M.W., Legrand A.P. (2007). Newly developed hybrid suture without lubricant: Noninvasive in vivo assessment of biocompatibility with multiparametric MR imaging. J. Investig. Surg..

[9] Karaca E., Hockenberger A.S. (2008). Analysis of the fracture morphology of polyamide, polyester, polypropylene, and silk sutures before and after implantation in vivo. J. Biomed. Mater. Res. Part B Appl. Biomater..

[10] Debbabi F., Gargoubi S., Hadj Ayed M.A., Abdessalem S.B. (2017). Development and characterization of antibacterial braided polyamide suture coated with chitosan-citric acid biopolymer. J. Biomater. Appl..

[11] Zhong W. (2008). Applications of Braided Structures in Medical Fields. Braided Structures and Composites: Production, Properties, Mechanics, and Technical Applications.

[12] Aibibu D., Hild M., Cherif C. (2016). An overview of braiding structure in medical textile: Fiber-based implants and tissue engineering. Advances in Braiding Technology.

[13] Rawal A., Sibal A., Saraswat H., Kumar V. (2015). Geometrically controlled tensile response of braided sutures. Mater. Sci. Eng. C.

[14] Sasaki Y., Tanaka Y., Ohtani A., Nakai A., Hamada H. Mechanical properties and fracture behavior of hybrid braided composite tube. Proceedings of the ICCM International Conferences on Composite Materials.

[15] Rawal A., Saraswat H., Sibal A. (2015). Tensile response of braided structures: A review. Text. Res. J..

[16] Omeroglu S. (2006). The effect of braiding parameters on the mechanical properties of braided ropes. Fibres. Text. East. Eur..

[17] Kyosev Y. (2015). Braiding Technology for Textiles.

[18] Del Rosso S., Iannucci L., Curtis P.T. (2015). Experimental investigation of the mechanical properties of dry microbraids and microbraid reinforced polymer composites. Compos. Struct..

[19] Basu A., Chatterjee S. (2013). A comparative study on primary wound closure by subcuticular suture using different suture materials with emphasis on complications, cosmesis and cost-effectiveness. Hell. J. Surg..

[20] Viju S., Thilagavathi G. (2012). Fabrication and characterization of silk braided sutures. Fibers Polym..

[21] Abellán D., Nart J., Pascual A., Cohen R.E., Sanz-Moliner J.D. (2016). Physical and mechanical evaluation of five suture materials on three knot configurations: An in vitro study. Polymers.

[22] Calabrese L., Fiore V., Bruzzaniti P., Scalici T., Valenza A. (2020). Pinned Hybrid Glass-Flax Composite Laminates Aged in Salt-Fog Environment: Mechanical Durability. Polymers.

[23] Calabrese L., Fiore V., Scalici T., Valenza A. (2019). Experimental assessment of the improved properties during aging of flax/glass hybrid composite laminates for marine applications. J. Appl. Polym. Sci..

[24] Swolfs Y., Gorbatikh L., Verpoest I. (2014). Fibre hybridization in polymer composites: A review. Compos. Part A Appl. Sci. Manuf..

[25] Sergi C., Tirillò J., Seghini M.C., Sarasini F., Fiore V., Scalici T. (2019). Durability of basalt/hemp hybrid thermoplastic composites. Polymers.

[26] Wu L., Wang W., Jiang Q., Xiang C., Lou C.W. (2019). Mechanical characterization and impact damage assessment of hybrid three-dimensional five-directional composites. Polymers.

[27] Rawal A., Kumar R., Saraswat H. (2012). Tensile mechanics of braided sutures. Text. Res. J..

[28] Heward A.G., Laing R.M., Carr D.J., Niven B.E. (2004). Tensile Performance of Nonsterile Suture Monofilaments Affected by Test Conditions. Text. Res. J..

[29] Abdessalem S.B., Debbabi F., Jedda H., Elmarzougui S., Mokhtar S. (2009). Tensile and Knot Performance of Polyester Braided Sutures. Text. Res. J..

[30] Abdessalem S.B., Jedda H., Skhiri S., Dahmen J., Boughamoura H. (2006). Improvement of mechanical performances of braided polyester sutures. Autex. Res. J..

[31] Debbabi F., Abdessalem S.B. (2018). Modelling and experimental investigation of mechanical performances of braided polyamide sutures. Indian J. Fibre Text. Res..

[32] Debbabi F., Abdessalem S.B. (2014). Simultaneous optimization of mechanical properties of braided polyethylene terephthalate suture subjected to hot-stretching treatment. J. Ind. Text..

[33] Debbabi F., Abdessalem S.B. (2015). Effect of manufacturing conditions on structural and handling properties of braided polyamide suture. J. Eng. Fiber. Fabr..

[34] Debbabi F., Abdessalem S.B. (2016). Impact of hot-stretching treatment on physical and mechanical properties of braided polyamide suture. Text. Res. J..

[35] Kalebek N.A., Konur E.E., Ozdinc O. (2016). Tensile and knot performance of polyester, silk, polypropylene and polydioxanone sutures. Tekst ve Muhendis.

[36] Sular V., Bulut Y. (2014). Tensile, knot, and detaching from needle performances of atraumatic surgical sutures. Int. J. Polym. Mater. Polym. Biomater..

[37] (2015). Standard Test Method for Tensile Properties of Yarns by the Single-Strand Method.

[38] Hristov K., Armstrong-Carroll E., Dunn M., Pastore C., Gowayed Y. (2004). Mechanical Behavior of Circular Hybrid Braids under Tensile Loads. Text. Res. J..

[39] (2006). Nonabsorbable Surgical Suture. United States Pharmacopeia and National Formulary (USP 29-NF 24).

[40] Naleway S.E., Lear W., Kruzic J.J., Maughan C.B. (2015). Mechanical properties of suture materials in general and cutaneous surgery. J. Biomed. Mater. Res. Part B Appl. Biomater..

[41] (2013). Standard Test Method for Breaking Strength and Elongation of Textile Webbing, Tape and Braided Material.

[42] Dabiryan H., Johari M.S., Bakhtiyari S., Eskandari E. (2017). Analysis of the tensile behavior of tubular braids using energy method, Part II: Experimental study. J. Text. Inst..

[43] (2008). Standard Practice for Conditioning and Testing Textiles.

[44] Fujita T. (2014). Choosing a better technique for midline abdominal closure. J. Am. Coll. Surg..

[45] Marom G., Fischer S., Tuler F.R., Wagner H.D. (1978). Hybrid effects in composites: Conditions for positive or negative effects versus rule-of-mixtures behaviour. J. Mater. Sci..

[46] Pan N., Chen K., Monego C.J., Backer S. (1998). The hybrid effects in hybrid fibre composites: Experimental study using twisted fibrous structures. Proc. R. Soc. Lond. A.

[47] Nanni F., Ruscito G., Forte G., Gusmano G. (2007). Design, manufacture and testing of self-sensing carbon fibre-glass fibre reinforced polymer rods. Smart Mater. Struct..

[48] Rajpurohit A., Joannès S., Singery V., Sanial P. (2020). Hybrid Effect in In-Plane Loading of Carbon/Glass Fibre Based Inter- and Intraply Hybrid Composites. J. Compos. Sci..

[49] Rana S., Zdraveva E., Pereira C., Fangueiro R., Correia A.G. (2014). Development of hybrid braided composite rods for reinforcement and health monitoring of structures. Sci. World J..

[50] Afzali Naniz M., Safar Johari M. (2019). An experimental study of the fiber hybridization effect on the mechanical performance of the 2D braided tubular composites. Mater. Res. Express.

[51] Jones K.D., DiBenedetto A.T. (1994). Fiber fracture in hybrid composite systems. Compos. Sci. Technol..

[52] Manders P.W., Bader M.G. (1981). The strength of hybrid glass/carbon fibre composites. J. Mater. Sci..

[53] Morton W.E., Hearle J.W.S. (2008). Physical Properties of Textile Fibres.

[54] Pytel A., Kiusalaas J. (2012). Mechanics of Materials.

[55] Chu C.-C., von Fraunhofer J.A., Greisler H.P. (1997). Wound Closure Biomaterials and Devices.

[56] Greenwald D., Shumway S., Albear P., Gottlieb L. (1994). Mechanical Comparison of 10 Suture Materials before and After in Vivo Incubation. J. Surg. Res..

[57] Karaca E., Hockenberger A.S., Yildiz H. (2005). Investigating Changes in Mechanical Properties and Tissue Reaction of Silk, Polyester, Polyamide, and Polypropylene Sutures in Vivo. Text. Res. J..

